# Pulmonary Langerhans cell histiocytosis presenting as an uncommon mass in the lung: A rare case report and literature review

**DOI:** 10.1002/ccr3.9342

**Published:** 2024-08-19

**Authors:** Tingxiu Zhang, Sheng Hu, Yue Teng, Zhiwei Li, Zhenliang Xiao, Lijie Ma

**Affiliations:** ^1^ Department of Pulmonary and Critical Care Medicine Chinese People's Liberation Army Western Theater General Hospital Chengdu Sichuan China

**Keywords:** chemotherapy, histiocytosis, Langerhans cells, pulmonary Langerhans cell histiocytosis

## Abstract

A comprehensive diagnostic approach, including immunohistochemistry, is crucial for confirming pulmonary Langerhans cell histiocytosis in adults. Individualized treatment with dynamically adjusted chemotherapy based on therapeutic response leads to significant absorption of lesions and symptom alleviation. Regular follow‐up and timely treatment adjustments according to the patient's condition are essential in managing this rare disease.

## 1 INTRODUCTION

Pulmonary Langerhans cell histiocytosis (PLCH) is a cystic lung disease and a distinct form of LCH.^1^ The condition predominantly affects men compared to women.^2^ Early symptoms of PLCH often include dyspnea, cough, sputum production, and chest pain.^3^ The pathogenic hallmark of PLCH is the proliferation of Langerhans cells in the alveolar and bronchial walls.^4^ These cells exhibit inflammatory responses, such as vasodilation, inflammatory infiltration, and fibrosis. This proliferative and inflammatory process can lead to damage to the alveolar and bronchial walls. Chronic consequences of this disorder include impaired lung function and dyspnea.

This article presents a retrospective analysis of clinical data from an adult patient diagnosed with PLCH, who was hospitalized and followed up at the General Hospital of Western Theater Command. The aim is to contribute to the understanding of PLCH by sharing clinical experience and insights gained from this case. This article presents a retrospective analysis of clinical data from an adult patient diagnosed with PLCH, who was hospitalized and treated at the General Hospital of Western Theater Command. By sharing the clinical experience and insights gained from this case, particularly in terms of the diagnostic process and treatment strategies, we aim to contribute to the understanding of PLCH and highlight the importance of individualized treatment approaches in managing this rare disease.

## 2 CASE REPORT

### 2.1 Background of the case

A 48‐year‐old female farmer, with no history of smoking, drinking, underlying diseases such as hypertension or diabetes, or family history of hereditary conditions, presented in good general health prior to the onset of her current symptoms. Due to limited mouth opening for 2 months, chest tightness and chest pain for 20 days, the patient was admitted to a nearby hospital in September of 2021 for further evaluation.

### 2.2 Treatment

On admission, the patient was found to have limited mouth opening, but no dysphagia, edema, fever, or pain in local tissues. She also reported no cough, expectoration, chest tightness, chest pain, or discomfort. The chest CT performed on October 8, 2021, revealed a higher density of masses in the right middle lobe, with friable boundaries. Despite receiving anti‐infective treatment at the regional hospital, the percutaneous lung biopsy findings showed extensive infiltration of the lung tissue by plasma cells, lymphocytes, and fibroblastic proliferation, which did not improve with the therapy.

**FIGURE 1:**
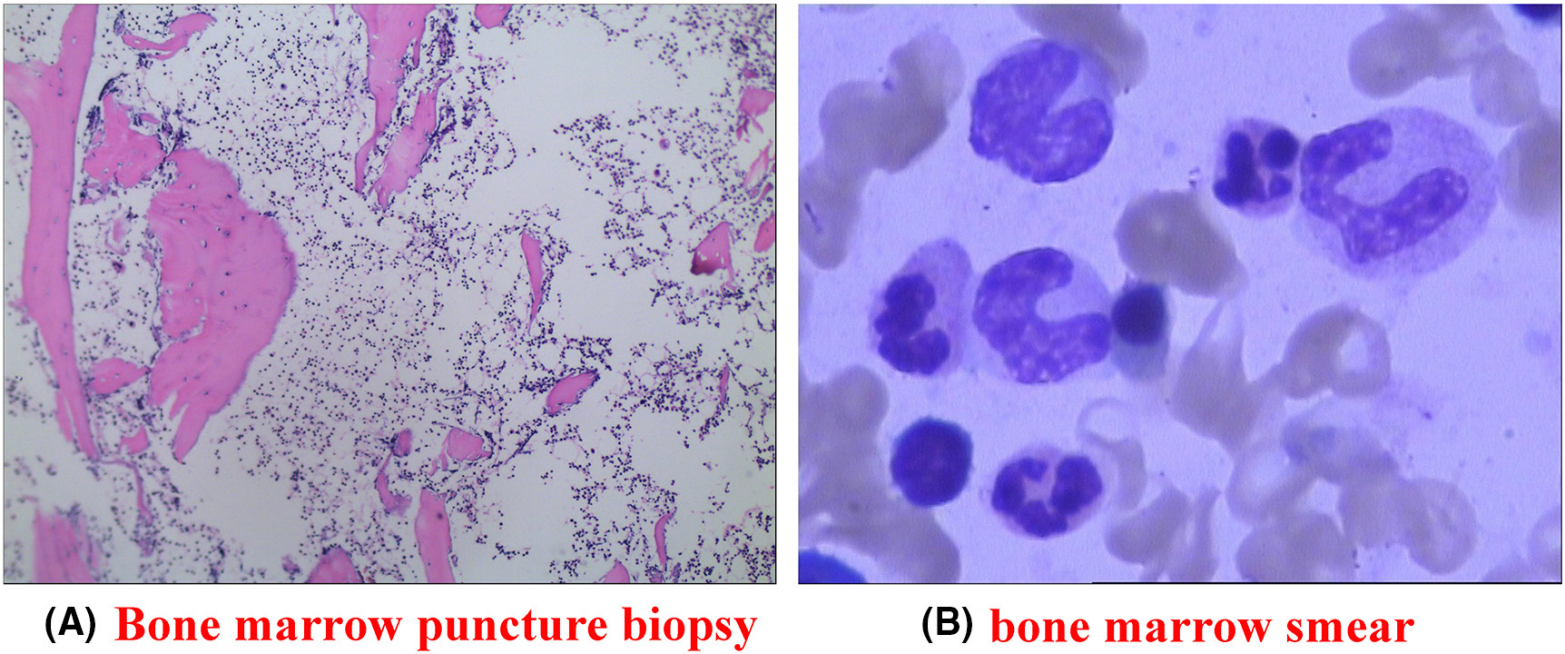
Bone marrow examination results after admission. (A) Bone marrow aspirate biopsy showing active proliferation. (B) Bone marrow smear examination revealing increased proliferating nucleated cells.

The patient was further treated in Sichuan Cancer Hospital for further management. A chest CT performed on November 1, 2021, revealed irregular soft tissue lesions occupying the right hilum and the lateral segment of the right middle lobe, along with several enlarged lymph nodes in the mediastinum and bilateral hila. Subsequently, a mediastinal lymph node biopsy (involving 1Ri and 11RS lymph nodes) was conducted. The histopathological examination of the biopsied tissues showed predominantly lymphocytes, histiocytes, and exudative tissue, with no definitive evidence of carcinomatous components. The drug sensitivity test indicated that the patient was sensitive to piperacillin sodium and tazobactam. Despite the administration of piperacillin sodium and tazobactam, an anti‐infective combination, the patient's symptoms, including limited mouth opening, did not improve. A follow‐up chest CT scan revealed that the lesion had increased in size compared to the previous examination. On December 10, 2021, a second lung biopsy was performed, and the pathological examination showed fibroblastic proliferation accompanied by giant cells and lymphocytic infiltration in the lung tissue. Concurrently, the patient developed soft tissue masses in the upper and left lower extremities, along with worsening chest tightness, pain, and discomfort, particularly upon deep inhalation. Moreover, a PET‐CT scan showed a mass shadow in the right lung accompanied by elevated glucose metabolism and obstructive pneumonia, suggestive of inflammatory lesions. The increased glucose metabolism observed in the lesions of the left maxillary sinus was more indicative of a benign or less invasive pathology. The presence of increased glucose metabolism and soft tissue edema in both upper extremities was consistent with inflammatory lesions. Due to the persistence of symptoms despite symptomatic treatment, the patient sought further medical attention at our hospital on December 29, 2021. Upon examination, a tender, warm, soft tissue mass measuring 5 cm × 6 cm was found in the cubital fossa of the left upper extremity. Similarly, an 8 cm × 5 cm soft tissue mass was observed in the left lower limb's calf, accompanied by an elevated local skin temperature and significant tenderness. Cardiac, pulmonary, and abdominal examinations revealed no abnormal findings. Upon admission, routine blood tests showed the following: leukocytes 21.08 × 10^9^/L, neutrophil ratio 88.8%, HGB 69 g/L, platelets 574 × 10^9^/L, CRP 276.88 mg/L, procalcitonin 0.95 ng/mL, and plasma D‐dimer 7.83 mg/L. The results of tests on the liver function were: Alanine transaminase (ALT) was 72.9 IU/L, aspartate transaminase (AST) 102.6 IU/L, and albumin 27.9 g/L. Tests for autoimmune antibodies, complement immunoglobulins, antineutrophil cytoplasmic antibodies (ANCA), urine Bence–Jones protein, renal function, and tumor markers were all within normal limits. Following admission, the patient developed fever with a temperature reaching 39°C. On January 5, 2022, a bone marrow aspirate biopsy was performed, which revealed active bone marrow tissue proliferation, characterized by marked granulocyte proliferation and increased eosinophil counts (Figure 1A). Bone marrow smear examination demonstrated an increased presence of proliferating nucleated cells. The percentage of neutrophilic segmented nucleated cells was markedly elevated at 20.5%, while plasma cells accounted for 2.5% of the total nucleated cell population (Figure 1B).

**FIGURE 2:**
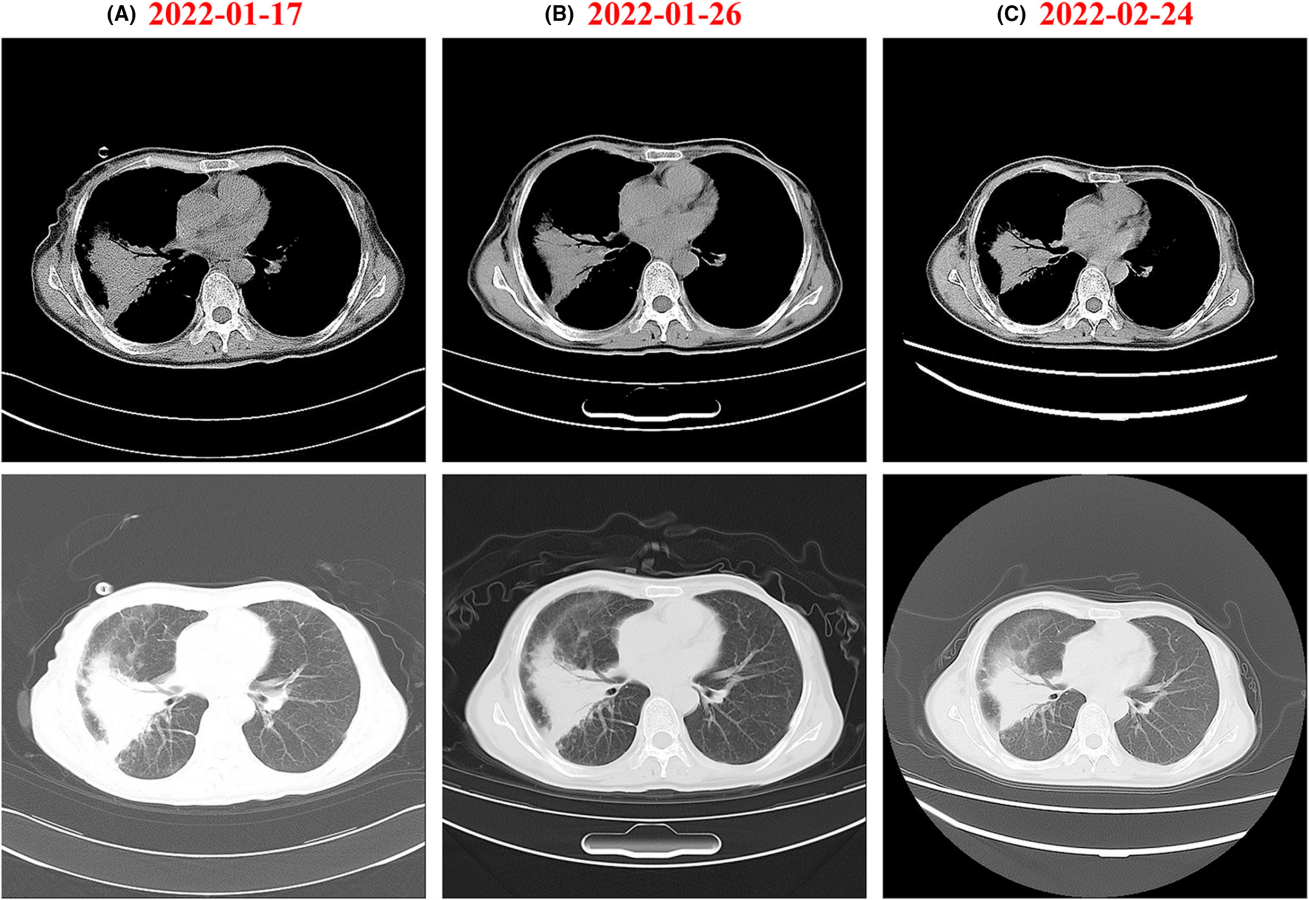
Chest CT scans before and after anti‐infective drug therapy. (A) CT scan performed on January 17, 2022, before treatment. (B) Follow‐up CT scan performed on January 26, 2022, after treatment. (C) Follow‐up CT scan performed on February 24, 2022.

A chest CT scan of the right lung performed on January 17, 2022, revealed findings suggestive of an infected lesion (Figure 2A). The patient underwent a 10‐day course of moxifloxacin and an 8‐day course of imipenem–cilastatin sodium for antimicrobial treatment. Follow‐up chest CT scans were performed on January 26 and February 24, 2022. Compared to the previous scans, these images revealed minimal absorption of the right lung lesion, suggesting a suboptimal response to the current treatment regimen (Figure 2B,C).

**FIGURE 3:**
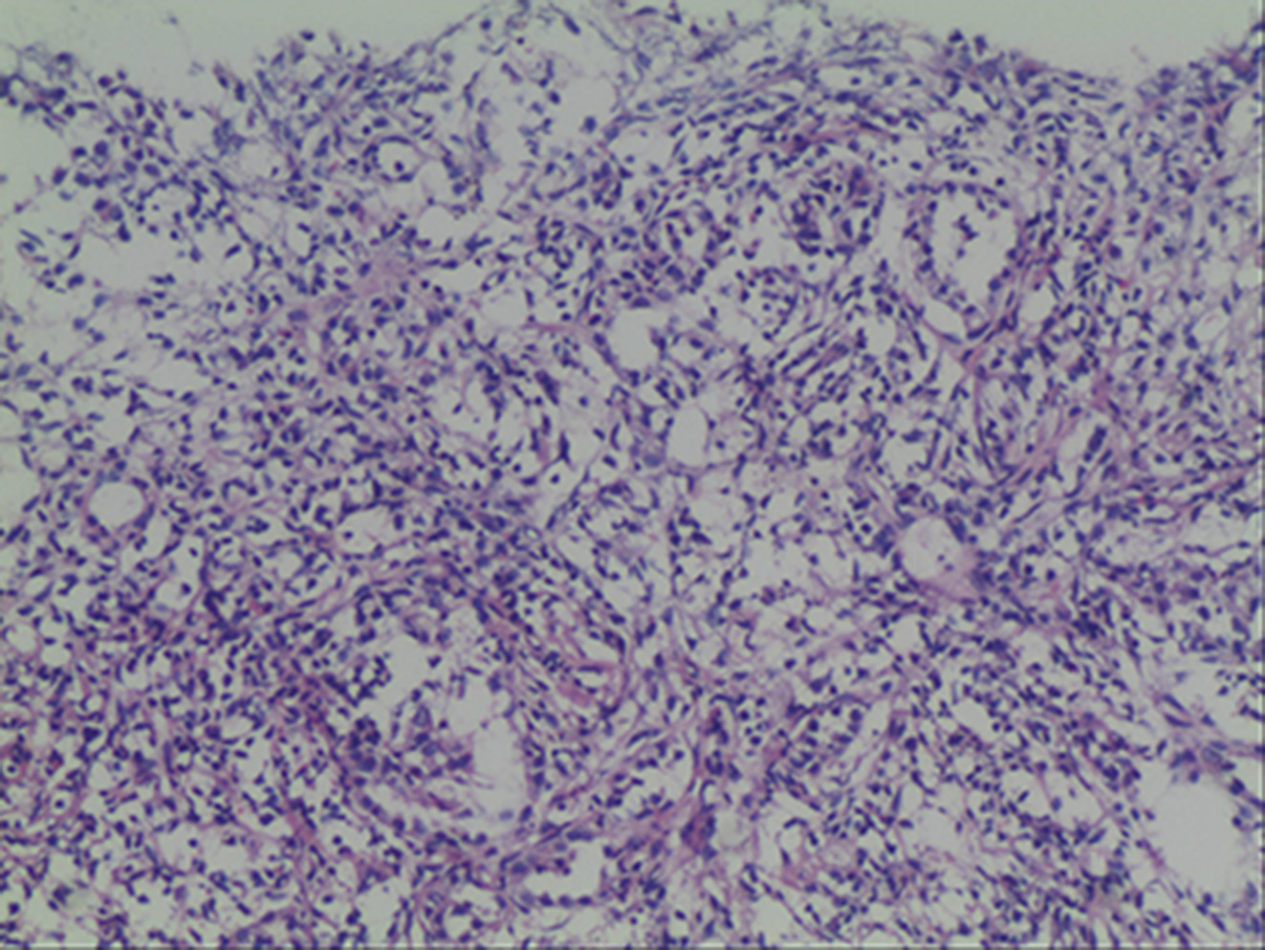
Lung biopsy results. A lung biopsy showed that there was a significant influx of inflammatory cells into the interstitial spaces, along with the formation of lymphoid nodules and aberrant hyperplasia of the remnant alveolar epithelium.

On March 14, 2022, a repeat lung biopsy was performed, which revealed minimal substantial changes in the lung tissue. The biopsy showed extensive interstitial inflammatory cell infiltration with lymphoid follicle formation and atypical hyperplasia of the residual alveolar epithelium (Figure 3). Immunohistochemistry staining results were as follows: epithelial markers CK5/6(−), CK7 (+), TTF (+); neuroendocrine markers CD56 (−), CgA (−), Syn (−); other markers TTF‐1 (diffuse +), NapsinA (focal+), P40 (−), and Ki67 (+, 5%).

Lung tissue samples were also sent to West China Hospital at Sichuan University for further analysis. The histopathological examination revealed atypical histiocytic proliferative lesions with a Langerhans cell phenotype, consistent with a diagnosis of PLCH. Immunohistochemical staining results were as follows: epithelial cells CK (+), CK5/6 (+); infiltrating single cells SMA (−), CD68 (+), CD163 (+), Des (−), S‐100 (+), CD1a (+), 1angerin (+); infiltrating lymphocytes CD3 fraction (+), CD20 fraction (+), CD30 (−) EBER (−), IG4 (individual, 0–7 per high magnification field), and no point mutation in exon 15 of the BRAF gene (V600E) was detected. Based on these findings, the patient was finally diagnosed with PLCH.

**FIGURE 4:**
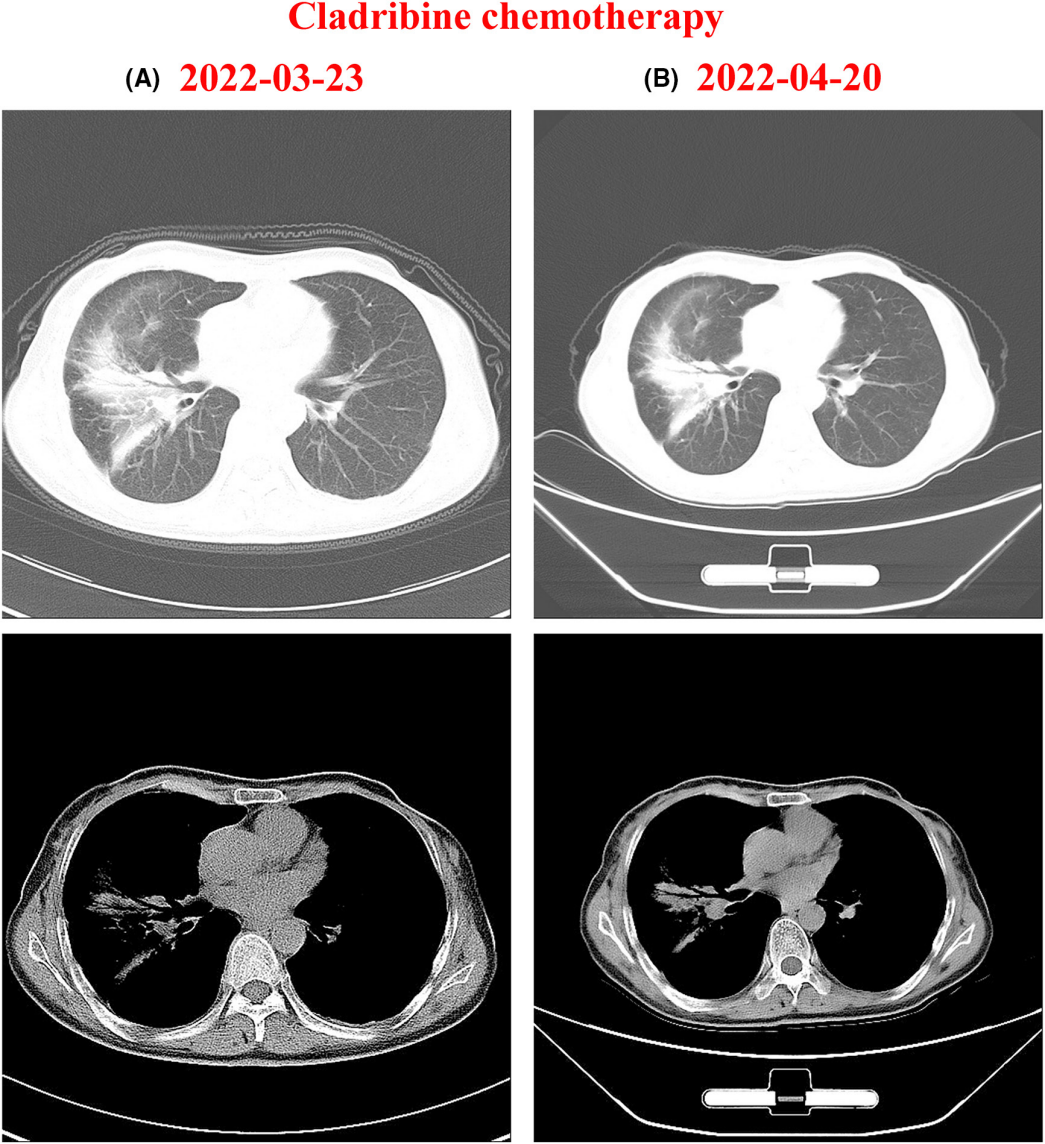
Chest CT scans before and after cladribine chemotherapy. (A) CT scan performed before cladribine treatment. (B) CT scan performed after cladribine treatment (10 mg, Days 1–5).

On March 24, 2022, the patient was transported to our hematology clinic and received cladribine 10 mg on Days 1–5 as chemotherapy. Chest CT scans were performed before (Figure 4A) and after (Figure 4B) the chemotherapy course. Although the scans showed slight absorption of the lesions compared to the pre‐chemotherapy images, the patient still experienced severe chest tightness and chest pain. During the course of cladribine monotherapy, the patient was also administered oral prednisone acetate tablets 100 mg daily as a systemic corticosteroid therapy. From May 24, 2022, to September 2, 2022, the chemotherapy regimen was adjusted to the cyclophosphamide, hydroxydaunorubicin, oncovin, prednisone, and etoposide phosphate (CHOP‐E) regimen based on the patient's condition. The CHOP‐E regimen, administered in four cycles with a 21‐day interval between each cycle, consisted of cyclophosphamide 1.1 g, hydroxydaunorubicin 75 mg, and oncovin 4 mg on Day 1; etoposide 150 mg on Days 2–4; and prednisone 100 mg on Days 1–5. In addition to the CHOP‐E regimen, the patient received continuous systemic corticosteroid therapy with oral prednisone acetate tablets (100 mg daily) throughout the chemotherapy cycles.

**FIGURE 5:**
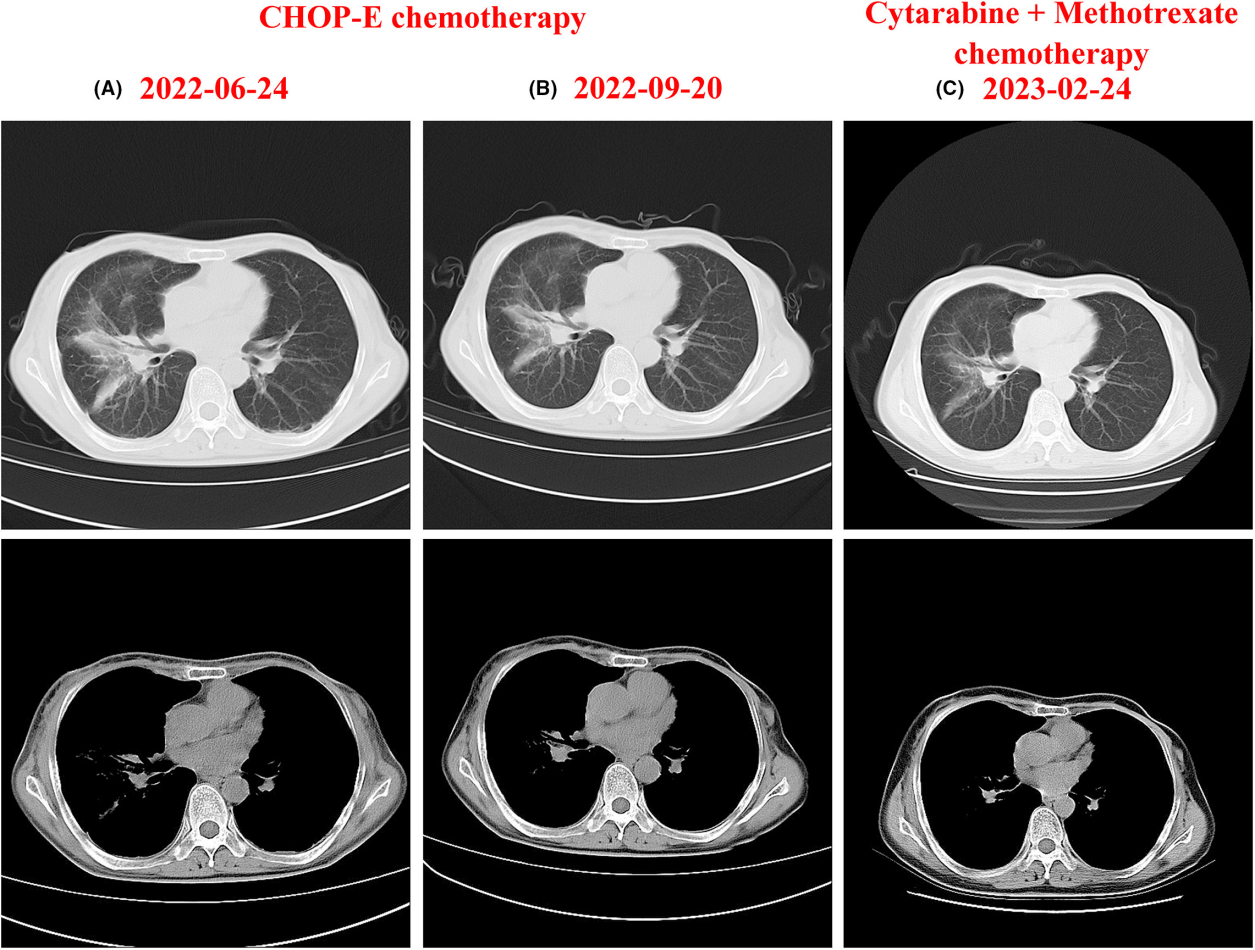
Chest CT scans following CHOP‐E and cytarabine + methotrexate chemotherapy regimens. (A) CT scan after one cycle of CHOP‐E chemotherapy. (B) CT scan after four cycles of CHOP‐E chemotherapy. (C) CT scan after two cycles of cytarabine (150 mg, Days 1–5) and methotrexate (1500 mg, Day 1) chemotherapy.

Chest CT scan performed after the first and fourth of CHOP‐E chemotherapy reveled that some PLCH lesions remained active following treatment. This was evidenced by local bone destruction and nodules in the left maxillary sinus floor wall, abnormal FDG metabolism, and a solitary enlarged lymph node at level II of the left neck (Figure 5A,B). In response to these findings, the patient's chemotherapy regimen was adjusted to cytarabine 150 mg on Days 1–5 and methotrexate 1500 mg on Day 1, for two cycles starting from November 10, 2022, through February 2023. Concurrently with the cytarabine and methotrexate chemotherapy, the patient was maintained on oral prednisone acetate tablets 100 mg daily as a systemic corticosteroid therapy. A follow‐up chest CT examination after this treatment showed significant absorption of the lesions, and the patient reported alleviation of chest tightness and chest pain (Figure 5C).

## 3 DISCUSSION

PLCH is a subtype of LCH, characterized by the abnormal proliferation and accumulation of Langerhans cells in the lungs.^5^ Chest X‐rays and CT scans are the gold standards for diagnosing PLCH.^6^ The hallmark of PLCH on CT scans is the presence of numerous small cysts in the lung parenchyma, which may appear isolated or coalesced. Currently, there are no established clinical guidelines for the diagnosis or treatment of PLCH, and its underlying pathophysiology remains poorly understood.

Due to the similarity of its symptoms to those of other pulmonary diseases, PLCH is often misdiagnosed or left untreated. Accurate diagnosis necessitates taking into account the patient's history, performing a comprehensive physical examination and pulmonary function test, and analyzing the results of any applicable imaging modalities. Typical clinical symptoms and immunohistochemistry data are crucial in making a diagnosis of PLCH. Most Langerhans cells were big and oval under a light microscope, and some had nuclei with a “coffee bean” nuclear groove.^7^ Positive results for the immunohistochemistry markers CD1a, CD207 (Langerin), and S100 were required for a definitive diagnosis.^8^, ^9^ Imaging studies of this patient's lungs revealed predominantly mass lesions, lacking the typical cystic and interstitial changes commonly observed in PLCH. Following the failure of initial empirical anti‐infective therapy to improve the patient's condition, multiple percutaneous lung biopsies were performed, which demonstrated plasma cell and lymphocyte infiltration, a characteristic feature of atypical histiocytosis. The patient's final immunohistochemistry results revealed positivity for S100, CD1a, Langerin, and CD20, which were consistent with the immunohistochemical diagnostic criteria for PLCH, thereby confirming the diagnosis.

Symptomatic relief, inflammation reduction, and enhanced lung function are the targets of PLCH treatment. Early diagnosis is crucial in the treatment of PLCH. Patients with mild symptoms still require regular monitoring of their pulmonary function and imaging studies to assess disease progression. For those experiencing severe symptoms, pharmacological interventions may be necessary. Treatment options for illness management and symptom alleviation may include glucocorticoids,^10^ chemotherapeutic medications,^11^, ^12^ and immunomodulatory drugs,^13^, ^14^ depending on the patient's state and the findings of the pathological investigation.

In this case, cladribine monotherapy was initially administered to the patient. After 1 month of treatment, a chest CT scan revealed a slight reduction in the lesion size. However, the patient's chest tightness and chest pain did not show significant improvement. Consequently, the chemotherapy regimen was further modified to the CHOP‐E protocol. Following this treatment, some lesions remained active, prompting a further adjustment of the chemotherapy regimen to a combination of cytarabine and methotrexate. Subsequent chest CT scans demonstrated significant absorption of the lesions, which correlated with the alleviation of the patient's chest tightness and pain. These findings highlight the importance of implementing personalized treatment approaches, as they led to a marked improvement in the patient's clinical outcomes.

Surgical treatment is an alternative treatment for PLCH. Surgical treatment is usually performed by means of pulmonary resection or lung transplantation.^15^ Pulmonary resection is an effective treatment that can relieve symptoms and manage the condition by removing the affected lung tissue. Lung transplantation is a more extreme treatment and is usually reserved for patients with severe PLCH.

## 4 CONCLUSIONS

In conclusion, adult PLCH is a rare disorder characterized by the abnormal proliferation and infiltration of Langerhans cells in the lungs. The wide variability in clinical manifestations and signs, which are dependent on the extent and location of the affected lung tissue, often leads to misdiagnosis. This case report highlights the importance of a comprehensive diagnostic approach, including immunohistochemical examination, in confirming the diagnosis of PLCH. The key clinical message of this report is that the management of adult PLCH requires a highly individualized approach tailored to each patient's specific presentation. In this case, the patient received multiple chemotherapy regimens, which were dynamically adjusted according to the therapeutic response, resulting in significant absorption of the lesions and alleviation of symptoms. Furthermore, regular follow‐up and timely adjustments to the treatment plan based on the patient's condition are crucial in managing this rare disease. As our understanding of the pathophysiology and management of PLCH continues to expand, it is anticipated that more therapeutic strategies and protocols will be developed and applied to the treatment of this disease in the future, ultimately improving patient outcomes.

## AUTHOR CONTRIBUTIONS

**Tingxiu Zhang:** Conceptualization; data curation; investigation; methodology; project administration; writing – original draft. **Sheng Hu:** Data curation; formal analysis; writing – original draft. **Yue Teng:** Data curation; formal analysis; writing – review and editing. **Zhiwei Li:** Investigation; writing – review and editing. **Zhenliang Xiao:** Supervision; writing – review and editing. **Lijie Ma:** Conceptualization; data curation; funding acquisition; methodology; project administration; software; visualization; writing – original draft; writing – review and editing.

## FUNDING INFORMATION

The paper was supported by project “FUNDC1‐mediated mitophagy in pulmonary arterial hypertension (41C416AD).”

## CONFLICT OF INTEREST STATEMENT

The authors declare no conflicts of interest.

## ETHICS STATEMENT

Ethical approval to report this case was obtained from Ethics Committee of Western Theater Command General Hospital of PLA (approval number: 2022ky016‐1).

## CONSENT

Written informed consent was obtained from the patient to publish this report in accordance with the journal's patient consent policy.

## Data Availability

The data used in this study are available from the corresponding author on reasonable request.
